# Immunotherapy with internally inactivated virus loaded dendritic cells boosts cellular immunity but does not affect feline immunodeficiency virus infection course

**DOI:** 10.1186/1742-4690-5-33

**Published:** 2008-04-17

**Authors:** Giulia Freer, Donatella Matteucci, Paola Mazzetti, Francesca Tarabella, Enrica Ricci, Leonia Bozzacco, Antonio Merico, Mauro Pistello, Luca Ceccherini-Nelli, Mauro Bendinelli

**Affiliations:** 1 Retrovirus Center and Virology Section, Department of Experimental Pathology, University of Pisa, Via del Brennero 2, I-56127 Pisa, Italy

## Abstract

Immunotherapy of feline immunodeficiency virus (FIV)-infected cats with monocyte-derived dendritic cells (MDCs) loaded with aldrithiol-2 (AT2)-inactivated homologous FIV was performed. Although FIV-specific lymphoproliferative responses were markedly increased, viral loads and CD4^+ ^T cell depletion were unaffected, thus indicating that boosting antiviral cell-mediated immunity may not suffice to modify infection course appreciably.

## Findings

Cell-mediated immune responses involving polyfunctional CD8^+ ^and CD4^+ ^T cells are considered pivotal in the control of human immunodeficiency virus (HIV)-1 infection [1]. Dendritic cells (DCs) are the only antigen-presenting cells that are able to present exogenous antigens to both helper and cytotoxic T cells; in order to do so, DCs must undergo maturation after antigen uptake, a process that can be started by cytokines and microbial products like lipopolysaccharide (LPS) [2,3]. Early clinical trials exploring autologous DCs that were loaded with antigens ex vivo to induce T-cell responses have provided proof of principle that DCs might be exploited in the therapy and/or prevention of various types of disease [4]. Recently, DCs have been also tested in the immunotherapy of HIV-1 and simian immunodeficiency virus infections [5,6]; in particular, Lu and colleagues have reported that stimulating the immune system with autologous monocyte-derived DCs pulsed with AT2-inactivated whole HIV-1 is beneficial in the treatment of chronic HIV-1 infection [5].

FIV is a non-primate lentivirus that has long been studied as a model for HIV [7]. The infection it establishes in cats closely resembles human AIDS, causing progressive immune deficiency and allowing it to be considered one of the best models to test different strategies against HIV-1. We have recently tested vaccination of cats with autologous MDCs loaded with AT2-inactivated FIV and matured with LPS (FIV-MDCs): such an approach elicited very high proliferative responses against FIV and detectable antibody responses [8]. However, such vaccination did not result in reduced infection of cats upon viral challenge. Because memory T cells have different requirements and may behave differently from naïve T cells upon activation [9], the present study was carried out to assess whether a similar approach might boost memory immune responses against FIV and lead to changes in immunological and virological parameters in chronically infected cats. The local isolate FIV-M2 was selected for the study, because it has been passed a limited number of times in tissue culture and preserves pathogenetic and neutralization features typical of wild-type lentiviruses [10]. FIV-M2 used to load MDCs was produced in interleukin (IL)-2 dependent MBM cells [10] grown in 3% cat plasma and inactivated by incubation with AT2 at a 300 μM final concentration at 4°C for 2 h, as described [8]. The virus thus treated (AT2-FIV) was then concentrated and purified on sucrose gradient for 3 h at 15,000 × g. A single stock containing 800 μg/ml protein was used throughout the study.

FIV-MDCs used for immunotherapy were prepared from heparinized jugular venous blood obtained under light anesthesia, 7 days before each inoculum, as described [11]. Briefly, peripheral blood mononuclear cells (PBMCs) were washed in apyrogenic saline and resuspended in RPMI 1640 supplemented with 2 mM L-glutamine, 1% non-essential amino acids, and 50 μg/ml gentamycin. Nine × 10^6 ^cells/well were distributed in 6-well plates, and then 3% autologous plasma was added. After 24 h, non-adhering cells were washed away, and 1 ml of medium containing 3% autologous plasma, recombinant feline IL-4, 10 ng/ml, and recombinant feline granulocyte-macrophage colony stimulating factor (R&D Systems, Minneapolis, MN), 50 ng/ml, were added. Every other day, fresh cytokines were added and, at day 5 of culture, MDCs were incubated with 80 μg/ml AT2-FIV at 37°C in 5% CO_2 _for 2 h. This antigen dose was 4 times the one used to load MDCs in our previous vaccine experiment mentioned above [8], in an attempt to maximize efficacy. Next, immediately after loading with the FIV antigen, MDCs were induced to mature by exposing them to 10 ng/ml of LPS from *E. coli *0127:B8 (Sigma, St Louis, MO) for 48 h. FIV-MDCs were then checked for MHC class II and B7.1 expression and for the ability to activate mixed lymphocyte reactions in vitro, as previously reported [8]: they exhibited high expression of MHC class II and B7.1 compared to immature MDCs in FACS analysis, and a highly upregulated ability to turn on reactivity by allogeneic PBMCs (data not shown).

**Figure 1:**
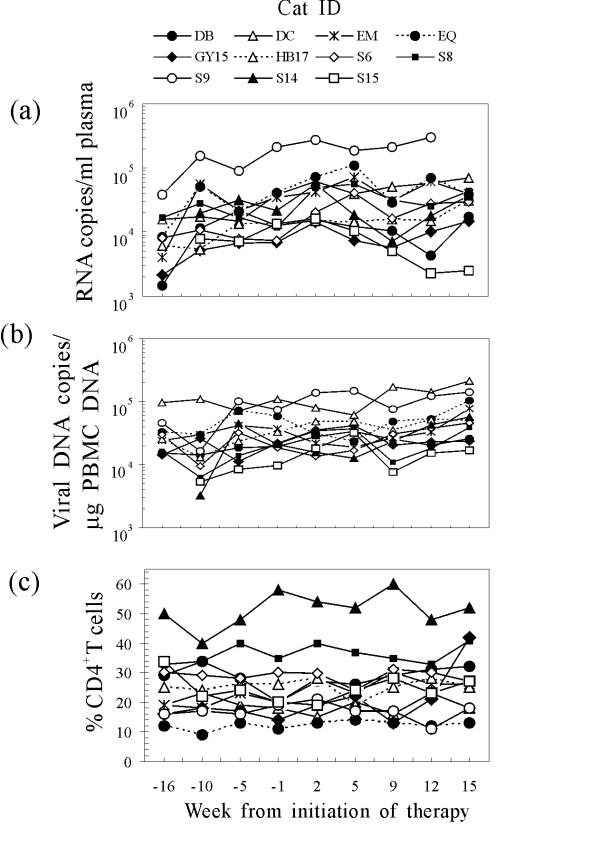
**Plasma viremia (a), proviral load in the PBMCs (b), and circulating CD4^+ ^T lymphocyte percentages (c) of the study cats at the times indicated relative to the first FIV-MDC inoculum.** CD4^+ ^T cells were monitored by flow cytometry in peripheral blood by direct staining with anti-feline CD4-PE (clone vpg34, AbD Serotec, Raleigh, NC) for 30 min as previously described (8). Symbols represent individual animals. Arrows indicate the times of FIV-MDC inoculation.

**Figure 2:**
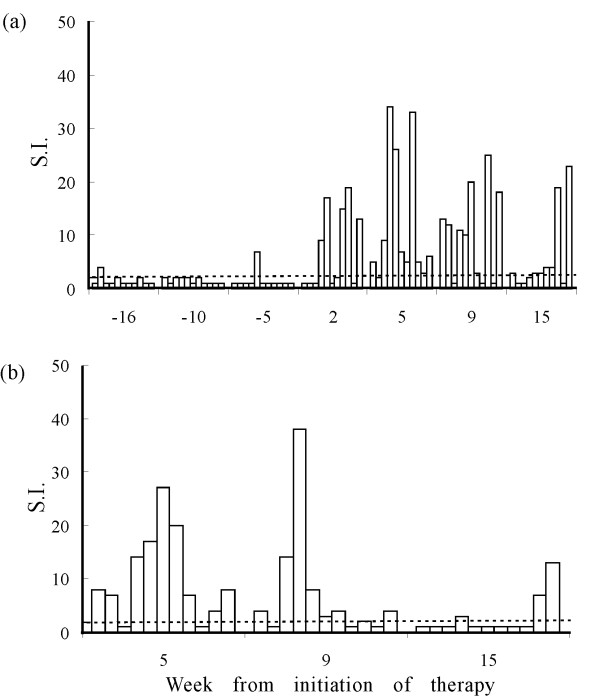
**Virus-specific lymphoproliferative activity in the study cats.** At the times indicated relative to the first FIV-MDC inoculum, PBMCs were exposed to intact FIV-M2 virions (a) or to pooled FIV-M2 Gag oligopeptides (b) and then examined for ^3^H-thymidine incorporation. Columns represent the stimulation index (S.I.) of individual animals, which was considered positive when above threshold dotted line (S.I. > 2). All cats responded to the nonspecific stimulus Concavalin A with counts per minute ranging from 10,000 to 38,000 counts per minute, while background proliferation in medium alone ranged between 100 and 1,100. At each sampling point, the animals are represented in the same order as shown in Figure 1.

For immunotherapy, MDCs were generated, loaded and, 2 days after exposure to antigen and LPS, reinjected into cats 3 times at 1-month intervals. In order to generate an in situ environment improving DC maturation [12,13], 20 min before FIV-MDCs were injected, two skin sites located on each thigh, close to the popliteal lymph nodes, were shaved and pretreated with the Toll-like receptor-7 agonist imiquimod (Aldara^® ^cream, Laboratoires 3 M Santé, Pontoise, France), that is also active for cats [14]. All FIV-MDCs obtained (Table 1) were resuspended in a final volume of 1 ml of saline, and 0.5 ml were injected subcutaneously at each site. Eleven specific-pathogen-free female cats, bought from IFFA Credo (L'Arbresle, France) and kept in our climate-controlled animal facility under conditions required by the European Community Law, were enrolled for this study. They had been infected intravenously with FIV-M2 [10] between 1 and 7 years before (Table 1) and, for at least 16 weeks before treatment, had exhibited essentially stable viral burdens in peripheral blood and, as expected, generally low CD4^+ ^T cell percentages (Figure 1). FIV-specific PBMC proliferative activity was determined by incubating 1.5 × 10^5 ^PBMCs in 96-well plates in 200 μl medium supplemented with 10% human serum with either 1 μg/well of purified sonicated FIV-M2 or no stimulus for 4 days, and counting after addition of ^3^H-thymidine (GE Healthcare, Milan, Italy) for 18 h. Results are reported as stimulation index (S.I.), that is the ratio of radioactivity incorporated by test PBMCs in the presence or absence of antigen, which was considered positive when >2 [10]. As shown by Figure 2a, prior to treatment, most animals tested negative in this assay; exceptions were cat GY, that had an S.I. of 7 at week -5, and cat DC, that had an S.I. of 4 at week -16. Already 2 weeks after the first FIV-MDC inoculum, 5 cats had markedly enhanced lymphoproliferative responses to FIV, most of which peaked at 5 weeks, when 10 of the 11 cats responded to therapy. Previous experiments in which cats immunized with whole uninfected MBM cells (where the virus used throughout the present study was produced) failed to generate immune responses that could be detected with the same assay used here, indicated that reactivity to cell components incorporated in the FIV virions did not contribute significantly to proliferation [10]. However, to confirm that proliferation was truly FIV specific, we also determined reactivity of PBMCs to the viral Gag. To this aim, a library of 58 15-mer overlapping peptides covering the entire FIV-M2 Gag p24 region, staggered by 4 amino acids, was resuspended at 1 mg/ml of each peptide in 100% DMSO and used at 5 μg/ml in the same assay. The results (Figure 2b) confirmed that proliferation was indeed FIV specific. Again, maximum reactivity was seen at week 5 post treatment, was still present in a good proportion of cats at week 9, and in a few cats at week 15. In contrast to cell mediated immunity, total binding antibody responses, determined by enzyme linked immunosorbent assays against purified FIV-M2 [10], turned out to be unaffected by therapy, in that plasma samples from experimental cats showed the same titers before (Table 1) and after treatment (not shown).

**Table 1:** Numbers of FIV-DCs inoculated per immunization into each experimental cat.

Cat ID	Years of infection	FIV-binding antibody titer^a^	FIV-DCs reinoculated (× 10^6^)			
			
			Inoculum			
				
			1st	2nd	3rd	Total
DB	7	3200	2	2	2	6
DC	7	3200	-	0.5	1.5	2
EM	3	800	3	3	1	7
EQ	3	3200	3	2	3	8
S6	7	6400	0.5	2	0.5	3
S8	7	3200	1.5	1	1	3.5
S9	7	6400	1	-	0.5	1.5
S14	7	800	1.5	0.5	0.5	2.5
S15	7	800	0.5	2	1	3.5
GY15	1	400	1.5	2.5	3	7
HB17	1	400	4.5	2	2	8.5

^a ^Titers are expressed as reciprocals of the highest serum dilutions that gave optical density readings at least threefold higher than the average values obtained with 10 control FIV-negative sera plus 3 times the standard deviation. Titers remained constant throughout the observation period.

^b ^FIV-MDCs were generated from each individual cat as described in the text. On the days of treatment, they were harvested, counted and reinjected into the corresponding donor cats. Numbers represent live cells, as evaluated by trypan blue exclusion.

To determine the effect of immunotherapy on FIV infection, the viral burden in the peripheral blood of experimental cats was measured at the time intervals shown in Figure 1 up to 15 weeks after the initiation of treatment. Plasma viremia was determined on RNA extracted from plasma samples by reverse transcription TaqMan polymerase chain reaction (TM-PCR). Proviral load in PBMCs was determined on PBMC DNA by TM-PCR. Sensitivities of the assays were 200 copies/ml of plasma and 100 copies/μg of genomic DNA, respectively [15]. As can be seen in Fig 1, cat identified as S15 exhibited a dramatic decrease in plasma viremia, but all the others remained at the same levels as before therapy. On the other hand, proviral DNA and CD4^+ ^T cell percentages invariably underwent no appreciable changes (Fig 1b and 1c).

The goal of vaccines in the therapy of viral diseases is the induction of protective immune responses able to control ongoing viral infection. Immunotherapy with AT2-FIV loaded MDCs did elicit a sharp and marked increase in FIV-specific PBMC proliferation. Although such an assay may be a crude indicator of the actual state of T cells involved in protection, we considered it as good as any other, especially in the light of many lentiviral vaccination experiments, dramatically confirmed by the recent failure of the STEP vaccine candidate to protect against HIV infection [16]. Indeed, although immunogenicity of the latter vaccine was demonstrated, mostly by ELISPOT, no protection was conferred to the vaccinees. In the present study, one cat (S15) out of the 11 enrolled exhibited reduced plasma viremia levels after FIV-MDC treatment, in parallel with relatively high lymphoproliferative response throughout the observation period. The significance of this finding, however, is questionable since the levels of viral DNA in the PBMCs and of circulating CD4^+ ^T cells remained unchanged. For all other cats, immunotherapy showed no beneficial effects, at least as detectable by the parameters monitored. This is in contrast with the afore mentioned studies in primates performed by Lu et al., since these workers reported significant viral load decreases in a high proportion of the subjects treated beginning soon after initiation of therapy [5,6].

The fact that total anti-FIV antibodies remained constant in titers throughout the observation period in our study came to no surprise, firstly because we had observed relatively low antibody responses in cats vaccinated with FIV-MDCs [8], secondly because it has been observed also in the immunotherapy of HIV patients by Lu and colleagues [6]. This issue, however, might be key to explaining why immunotherapy did not lead to a decrease in virus titers, since recent evidence has shown that potent immune activation of the cellular arm is not sufficient to reduce virus persistence in the absence of virus-specific neutralizing antibodies, but rather it might favor the formation of "sanctuaries" where viruses might persist undisturbed [17]. Alternatively, since the study cats had been infected for at least 1 year when they were treated, the virus they harbored might have diverged substantially in the T epitopes important for infection control from the virus used for preparing the FIV-MDCs, which was the same used for cat infection [18]. Another issue might be the low numbers of antigen-loaded MDCs that were delivered to the cats: although we did make an effort to obtain as many MDCs at each inoculation time as possible, their numbers were always much lower than the ones reported in studies of this kind with primates [5,6]. However, as we previously noticed [8] and in agreement with other studies [6,19], there was little or no correlation between the total amount of MDCs injected and the size of FIV-specific PBMC proliferation these elicited.

To sum up, immunotherapy of FIV infected cats does not seem to be an encouraging approach as currently performed. Should the data in the SIV/HIV systems by Lu et al [5,6] be confirmed independently, the question why FIV-loaded MDCs do not elicit beneficial effects in infected cats remains open. Differences between the present study and the ones mentioned above include the numbers of MDCs injected and the way maturation of MDCs was achieved, with LPS in the present study and with cytokines in the ones by Lu et al. Possible future improvements might include the development of effective strategies for in vivo targeting of antigen to DCs [20], but this will require targeting molecules that still need to be characterized in the cat model.

## Competing interests

The authors declare that they have no competing interests.

## Authors' contributions

GF and DM contributed equally to designing experiments and writing the manuscript. PM, FT, LB performed cellular biology and immunology experiments, AM performed animal procedures, ER, MP and LCN did molecular biology experiments, MB contributed to experimental design and revising the manuscript critically. All authors read and approved the final manuscript.
